# One year follow-up of patients with refractory angina pectoris treated with enhanced external counterpulsation

**DOI:** 10.1186/1471-2261-6-28

**Published:** 2006-06-15

**Authors:** Thomas Pettersson, Susanne Bondesson, Diodor Cojocaru, Ola Ohlsson, Angelica Wackenfors, Lars Edvinsson

**Affiliations:** 1 Department of Medicine, Kristianstad, Sweden; 2 Department of Emergency Medicine, Clinical Sciences Lund, Lund University, Sweden

## Abstract

**Background:**

Enhanced external counterpulsation (EECP) is a non-invasive technique that has been shown to be effective in reducing both angina and myocardial ischemia in patients not responding to medical therapy and without revascularization alternatives. The aim of the present study was to assess the long-term outcome of EECP treatment at a Scandinavian centre, in relieving angina in patients with chronic refractory angina pectoris.

**Methods:**

55 patients were treated with EECP. Canadian cardiovascular society (CCS) class, antianginal medication and adverse clinical events were collected prior to EECP, at the end of the treatment, and at six and 12 months after EECP treatment. Clinical signs and symptoms were recorded.

**Results:**

EECP treatment significantly improved the CCS class in 79 ± 6% of the patients with chronic angina pectoris (*p *< 0.001). The reduction in CCS angina class was seen in patients with CCS class III and IV and persisted 12 months after EECP treatment. There was no significant relief in angina in patients with CCS class II prior to EECP treatment. 73 ± 7% of the patients with a reduction in CCS class after EECP treatment improved one CCS class, and 22 ± 7% of the patients improved two CCS classes. The improvement of two CCS classes could progress over a six months period and tended to be more prominent in patients with CCS class IV. In accordance with the reduction in CCS classes there was a significant decrease in the weekly nitroglycerin usage (*p *< 0.05).

**Conclusion:**

The results from the present study show that EECP is a safe treatment for highly symptomatic patients with refractory angina. The beneficial effects were sustained during a 12-months follow-up period.

## Background

Refractory angina pectoris is a clinical diagnosis which is characterized by chronic angina due to coronary artery insufficiency in patients who are refractory to conventional forms of treatment [1]. Treatment of coronary artery disease consists of pharmacological interventions and invasive actions such as percutaneous coronary interventions (PCI) and coronary bypass grafting (CABG). In spite of these generally successful means of treatment the number of patients with severe symptomatic ischemic chest pain has increased [2]. It has been reported that up to 15% of patients with angina pectoris meet the criteria for refractory angina [3]. This is a significant clinical problem and the search for alternative therapies have yielded some new treatments such as Spinal Cord Stimulation (SCS) [4,5], left stellate ganglion blockade [2,6], thoracic epidural anesthesia [2,7] and Enhanced External Counter Pulsation (EECP) [8]. Currently, EECP therapy is one of the most promising treatments for relieving angina and has been shown to improve exercise tolerance in patients with symptoms of stable angina pectoris [9].

EECP is a non-invasive counterpulsation technique, which uses three sets of pneumatic cuffs wrapped around the lower extremities. The cuffs are inflated sequentially at the onset of diastole, producing aortic counter pulsation, diastolic augmentation, and increased venous return. At the onset of systole, the external pressure in the cuffs is released, producing a decrease in systolic pressure. The hemodynamic effects are similar to intra-aortic balloon pumping (IABP). In contrast to IABP, EECP provides long-lasting increase in coronary blood flow [10,11]. A treatment procedure involves 1 to 2 hours/day for a total of 35 hours of therapy. Several studies have shown patient improvement with lowering in Canadian Cardiovascular Society Classification (CCS) [12,13]. In addition to relieving myocardial ischemia, EECP is associated with improved quality of life [13,14].

The aim of the present study was to evaluate the effect of EECP treatment at a Scandinavian centre on patients with refractory angina pectoris. The study was designed to examine the immediate, six months and 12 months follow-up effects on patients with severe refractory angina in whom multiple CABG and PCI have already been done and where further medical and surgical intervention were exhausted.

## Methods

### Patients in the study

55 patients, (47 male, 8 female, 45–89 years of age) with chronic stable refractory angina pectoris that were consecutively treated with EECP at the Kristianstad Hospital were included in this study. Eight patient experienced adverse events during the EECP treatment which resulted in termination of their treatment. These patients were not included in the follow-up investigations. The criteria for chronic stable refractory angina were defined by Mannheimer and colleagues in 2002 as "a chronic condition characterized by the presence of angina caused by coronary insufficiency in the presence of coronary artery disease which cannot be controlled by a combination of medical therapy, angioplasty and coronary bypass surgery. The presence of reversible myocardial ischemia should be clinically established to be the cause of the symptoms. Chronic is defined as a duration of more than 3 months " [1].

All patients had angiographically proven coronary stenosis (> 70%) in at least one major coronary artery and developed > 1 mm ST-segment depression or positive scintigraphic defects during exercise. For baseline characteristics and pharmacological treatment of the patients included in the follow-up study (47 patients), see Table 1 and 2. An informed consent was obtained from all patients included in the study. The study was performed in accordance with the Lund University Ethics Committeé.

**Table 1:** Baseline characteristics

Mean age, range (years)	66, 45–89
Gender (men/women)	40/7
	
*Co-existing disease*	
Heart failure	41%
Hypertension	45%
Diabetes mellitus	22%
	
*Coronary artery disease factors and revascularization status*	
CAD diagnosis (years; mean, range)	13, 1–35
Prior myocardial infarction	64%
Left ventricular ejection fraction	
EF ≥ 50%	59%
40% ≤ EF < 50%	30%
30% ≤ EF < 40%	9%
EF < 30%	2%
	
Prior PCI	62%
Prior CABG surgery	79%
Prior PCI and CABG surgery	49%
Angina CCS class (% of patients)	
I	0
II	11%
III	74%
IV	15%

CAD = Coronary Artery Disease, CABG = Coronary Artery Bypass Graft, PCI = Percutaneous Coronary Intervention, CCS = Canadian Cardiovascular Society Classification

**Table 2:** Pharmacological treatment

Medication	Baseline
β-blockers	89%
Ca^2+ ^antagonists	51%
Nitroglycerin	87%
**1–2 times/week**	**12%**
**3–7 times/week**	**22%**
**>7 time/week**	**66%**
Anticoagulants	6%
ACEI	45%
ARB	6%
Diuretics	30%
Insulin	9%
Statins	96%

ACEI = angiotensin converting enzyme inhibitor, ARB = angiotensin type 1 receptor blocker

### EECP treatment

The EECP device consists of three paired pneumatic cuffs applied to the lower extremities (Vasomedical, Westbury, New York, USA). The cuffs are inflated sequentially (applying 250–300 mmHg of external pressure) during diastole, returning blood from the legs to the central circulation, producing aortic diastolic augmentation and thus increasing both venous return and cardiac output. The cuffs are then deflated at end-diastole, reducing peripheral resistance and providing left ventricular unloading. Daily one hour treatment sessions are typically administered for a total treatment course of 35 hours.

### Data collection

Data on demographics, medical history, coronary disease status and medication were collected on patients before EECP treatment. No attempt was made to maintain current medication regimens throughout the study, although patients referred for EECP were considered "optimally medically managed". CCS class, antianginal medication use, and adverse clinical events were registered. Patients were interviewed by telephone six months after their last EECP treatment session, and 12 months thereafter to record anginal status and cardiac events.

### Calculation and statistics

All calculations and statistics were performed using GraphpadPrism 4.0. Statistical significance was accepted when *p *< 0.05, using student's *t*-test when comparing two groups and ANOVA with Dunnett's post hoc test when comparing more than two groups. Values are presented as means ± S.E.M.

## Results

**Figure 1:**
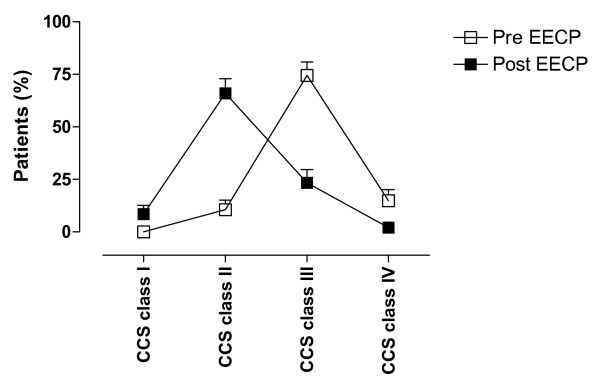
Overall changes in CCS class before (pre-EECP, □) and after (post-EECP, ■) EECP treatment. The figure shows a shift towards improved CCS class after EECP treatment. Values are calculated as percentage of total number of patients and are presented as mean ± SEM.

**Figure 2:**
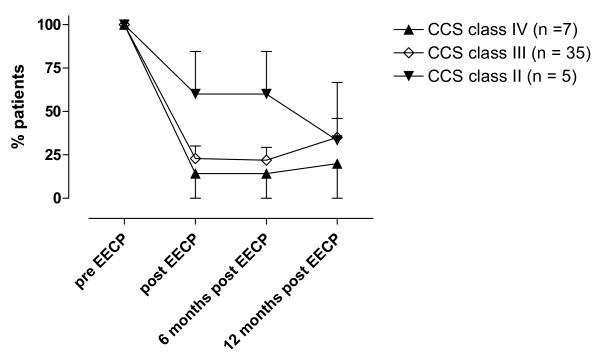
Changes in angina status over a 12-months period in patients with CCS class IV (**A**), III (**B**), and II (**C**) prior to EECP treatments. The figure shows percentage of patients in each CCS class before EECP treatment (100%) and how many (%) of these patients that still are in the same CCS class immediately, six months and 12 months after the treatment. n = number of patients in the CCS class before EECP treatment. All values were compared to pre-EECP values in each CCS class and are presented as mean ± SEM.

The total cohort completed 36.6 ± 0.5 hours of EECP treatment. EECP treatment improved the CCS class in 79% of the patients with chronically stable angina pectoris (Figure 1). The mean value of CCS classes prior to EECP treatment were significantly higher as compared to mean value after EECP treatment (3.0 ± 0.1 as compared to 2.2 ± 0.1, *p *< 0.001). The angina functional class did not change in 21% of the patients and importantly no patient changed to a higher CCS class directly after EECP treatment. Most patients (89%) were in CCS class III and IV pre-EECP treatment and 79% of these patients reduced their angina with at least one CCS class. The improvement in CCS class was significant in patients with CCS class III and IV and persisted six and 12 months after EECP treatment, while there was no such reduction in angina status in patients with CCS class II (Figure 2). Most patients improved one CCS class (73 ± 7%), thus 27 ± 7% of the patients improved two CCS classes and the beneficial effects were sustained at the 12-months follow-up. The improvement of two CCS classes tended to be more prominent in patients with CCS class IV prior to the EECP treatment and progressed over a six month period. 86% of the patients in CCS class IV prior to the EECP treatment improved in angina functional class. All of the patients that had improved two CCS classes had done this within six months after the treatment. During the follow-up period one patient died six month after EECP treatment in a myocardial infarction.

The weekly nitroglycerin usage was decreased after EECP treatment. 87 ± 5% of the patients used nitroglycerin before EECP treatment and 63 ± 7% used nitroglycerin after EECP treatment (*p *< 0.01). The other daily medication remained unaltered.

All patients underwent the initial phase of the EECP treatment without problems. However, during the EECP treatment period, adverse events were noted in eight cases which forced them to terminate their treatment (Table 3). They were not included in the follow-up investigations. One patient died after 15 treatment sessions. The death was considered as sudden death with no sign of worsening of the angina immediately before the death. One patient suffered from a myocardial infarction between treatment sessions nine and ten. The patient died in a myocardial infarction two weeks after termination of the EECP therapy. Two patients had increased chest pain and four patients had gastrointestinal problems.

**Table 3:** Adverse Effects

Patients (gender, age)	Number of sessions before termination	Cause of termination
Male, 50	12	Increased chest pain
Male, 84	15	Death in myocardial infarction
Female, 57	2	Emesis
Male, 58	6	Hiatus hernia
Male, 53	25	Colics of the bile system
Male, 77	9	Hemorrhoidal problems
Male, 74	25	Chest pain and minor myocardial ischemia
Male, 59	9	Death in myocardial infarction

## Discussion

The present study is the first long-term systematic follow-up study from a Scandinavian centre of consecutive patients treated with EECP for chronic stable refractory angina pectoris. The majority of the patients were men and showed a profile of extensive coronary artery disease, previous revascularizations and a poor quality of life. The patients were not available for further coronary revascularization and were on optimal pharmacological treatment. The medical regimen was not changed during the EECP treatment.

The results from the present study confirm that EECP treatment significantly reduces the CCS class in patients with chronic stable angina pectoris, which is in accordance with previous American studies [8,15-17]. It was noted that there was a significant decrease in the frequency of anginal episodes and nitroglycerin usage. EECP increases diastolic aortic pressure, reduces systolic pressure and enhances venous return, thus resulting in increased cardiac output [18]. However, the mechanisms by which these hemodynamic effects lead to a reduction of angina are poorly understood, although the effect is similar to IABP [11]. There is accumulating evidence suggesting that EECP treatment improves endothelial function, which may contribute to the clinical benefit [12]. EECP treatment is associated with an immediate increase in blood flow in multiple vascular beds including the coronary arterial circulation [11]. This increase in blood flow may result in increased endothelial shear stress [19], which enhances endothelial function by stimulating the release of the vasodilatory mediator nitric oxide and reduces the release of the vasocontractile endothelin-1 [18,20-22]. Furthermore, besides the release of metabolites from ischemic regions, an increase in endothelial shear stress is considered a major stimulus for collateral blood vessel development and recruitment [23]. This suggests that EECP treatment may exert its clinical beneficial effect by enhancement of coronary collateralization. EECP therapy has been associated with the release of angiogenic factors, such as vascular endothelial growth factor [23], basic fibroblast growth factor and hepatocyte growth factor [24].

The relief in CCS class was seen in patients with CCS class III and IV, while there was no beneficial effect in patients with CCS class II. Previous studies have shown a beneficial effect even in patients with mild angina [25]. The reason for the lack of effect in patient with CCS class II in the present study may be due to the limited number of patients in this group. These results indicate that the EECP treatment may be more effective in patients with the most disabling angina, which is in accordance with previous findings [26]. The reason for this is not known, although given the important role of shear stress for endothelial function, the shear stress forces may be stronger in patients with severe angina as compared to patients with mild angina [20,21]. Also, it might be easier for a patient to experience an improvement from CCS class IV to III, as compared to CCS class II to I, due the classification scale of the different angina functional classes.

73% of the patients who experience a beneficial effect of the EECP treatment improved one CCS class, and 27% of the patients improved two CCS classes. The relief of two CCS classes tended to progress over a period of six months and was more prominent in patients with CCS class IV prior to the EECP treatment. This delayed improvement in functional angina class has, to our knowledge, never been reported before. It is furthermore noteworthy that the improvement persisted in the 12-months follow-up. In a previous study by Masuda and colleagues it was shown that the plasma levels of nitric oxide is not increased immediately after completion of therapy but one month after [22]. One possible explanation to this delay may be an up-regulation of the endothelial nitric oxide synthase, the major source of endothelial nitric oxide [22]. This would result in a delay of improved endothelial function [27], and may explain the sustained effect of EECP treatment seen in the present study. Furthermore, the indication of EECP treatment promoting angiogenesis could also be an explanation to the delayed and persistent beneficial effect of the current treatment [28].

When stopping medical treatment or physical training no beneficial effect would be expected in a 12 months follow-up. Although, long-term effects after EECP treatment have been confirmed in the present study and in previous clinical [9,13,29] and observational studies [14,18]. The pathophysiological explanation for the long-term effects is not fully understood and need further studies. Thus, the initial improvement in CCS class after EECP therapy allows more physical activity [29], which may induce similar stimuli as EECP treatment [12].

### Limitation of the study

The present study is a follow-up report that does not include a control group, therefore a possible placebo effect can not be excluded. The improvement in CCS class could in such a case be a result of special attention of the patients during the follow-up and also statistically regression towards mean. The adverse events are in accordance with what is normally seen in this type of patients and there was no increase due to the EECP treatment. Thus, EECP therapy appears to be a promising alternative treatment to patients with severe refractory angina pectoris where medical treatment and surgical procedures are exhausted.

## Conclusion

The present study is the first to evaluate the effect of EECP treatment at a Scandinavian centre on patients with refractory angina pectoris. In summary, we found that EECP is a safe treatment for highly symptomatic patients with refractory angina. The effects were sustained in most of the patients at a 12-months follow-up. These results verify that the EECP treatment should be considered as an alternative treatment for patients with chronic refractory angina.

## Competing interests

The author(s) declare that they have no competing interests.

## Authors' contributions

TP was responsible for the treatment of the patients and was involved in initiating and designing the study and drafted the manuscript along with OO. SB and DD treated the patients and collected the data. AW was involved in analyzing the data and writing the manuscript. LE supervised the collection of data and writing of the final manuscript. All authors have read and approved the final manuscript.

## Pre-publication history

The pre-publication history for this paper can be accessed here:



## References

[B1] Mannheimer C, Camici P, Chester MR, Collins A, DeJongste M, Eliasson T, Follath F, Hellemans I, Herlitz J, Luscher T, Pasic M, Thelle D (2002). The problem of chronic refractory angina; report from the ESC Joint Study Group on the Treatment of Refractory Angina. Eur Heart J.

[B2] Yang EH, Barsness GW, Gersh BJ, Chandrasekaran K, Lerman A (2004). Current and future treatment strategies for refractory angina. Mayo Clin Proc.

[B3] Mannheimer C (1998). Therapeutic challenges of refractory angina
pectoris. In: XXth Congress of the European Society of. Cardiology, Vienna, Austria.

[B4] de Jongste MJ, Hautvast RW, Hillege HL, Lie KI (1994). Efficacy of spinal cord stimulation as adjuvant therapy for intractable angina pectoris: a prospective, randomized clinical study. Working Group on Neurocardiology. J Am Coll Cardiol.

[B5] Ekre O, Norrsell H, Wahrborg P, Eliasson T, Mannheimer C (2003). Temporary cessation of spinal cord stimulation in angina pectoris-effects on symptoms and evaluation of long-term effect determinants. Coron Artery Dis.

[B6] Chester M, Hammond C, Leach A (2000). Long-term benefits of stellate ganglion block in severe chronic refractory angina. Pain.

[B7] Richter A, Cederholm I, Jonasson L, Mucchiano C, Uchto M, Janerot-Sjoberg B (2002). Effect of thoracic epidural analgesia on refractory angina pectoris: long-term home self-treatment. J Cardiothorac Vasc Anesth.

[B8] Lawson WE, Hui JC, Soroff HS, Zheng ZS, Kayden DS, Sasvary D, Atkins H, Cohn PF (1992). Efficacy of enhanced external counterpulsation in the treatment of angina pectoris. Am J Cardiol.

[B9] Arora RR, Chou TM, Jain D, Fleishman B, Crawford L, McKiernan T, Nesto RW (1999). The multicenter study of enhanced external counterpulsation (MUST-EECP): effect of EECP on exercise-induced myocardial ischemia and anginal episodes. J Am Coll Cardiol.

[B10] Taguchi I, Ogawa K, Kanaya T, Matsuda R, Kuga H, Nakatsugawa M (2004). Effects of enhanced external counterpulsation on hemodynamics and its mechanism. Circ J.

[B11] Michaels AD, Accad M, Ports TA, Grossman W (2002). Left ventricular systolic unloading and augmentation of intracoronary pressure and Doppler flow during enhanced external counterpulsation. Circulation.

[B12] Bonetti PO, Holmes DRJ, Lerman A, Barsness GW (2003). Enhanced external counterpulsation for ischemic heart disease: what's behind the curtain?. J Am Coll Cardiol.

[B13] Michaels AD, Linnemeier G, Soran O, Kelsey SF, Kennard ED (2004). Two-year outcomes after enhanced external counterpulsation for stable angina pectoris (from the International EECP Patient Registry [IEPR]). Am J Cardiol.

[B14] Springer S, Fife A, Lawson W, Hui JC, Jandorf L, Cohn PF, Fricchione G (2001). Psychosocial effects of enhanced external counterpulsation in the angina patient: a second study. Psychosomatics.

[B15] Lawson WE, Hui JC, Lang G (2000). Treatment benefit in the enhanced external counterpulsation consortium. Cardiology.

[B16] Stys T, Lawson WE, Hui JC, Lang G, Liuzzo J, Cohn PF (2001). Acute hemodynamic effects and angina improvement with enhanced external counterpulsation. Angiology.

[B17] Barsness G, Feldman AM, Holmes DRJ, Holubkov R, Kelsey SF, Kennard ED (2001). The International EECP Patient Registry (IEPR): design, methods, baseline characteristics, and acute results. Clin Cardiol.

[B18] Barsness GW (2001). Enhanced external counterpulsation in unrevascularizable patients. Curr Interv Cardiol Rep.

[B19] Kern MJ, Aguirre FV, Tatineni S, Penick D, Serota H, Donohue T, Walter K (1993). Enhanced coronary blood flow velocity during intraaortic balloon counterpulsation in critically ill patients. J Am Coll Cardiol.

[B20] Kuchan MJ, Frangos JA (1993). Shear stress regulates endothelin-1 release via protein kinase C and cGMP in cultured endothelial cells. Am J Physiol.

[B21] Davies PF (1995). Flow-mediated endothelial mechanotransduction. Physiol Rev.

[B22] Masuda D, Nohara R, Hirai T, Kataoka K, Chen LG, Hosokawa R, Inubushi M, Tadamura E, Fujita M, Sasayama S (2001). Enhanced external counterpulsation improved myocardial perfusion and coronary flow reserve in patients with chronic stable angina; evaluation by(13)N-ammonia positron emission tomography. Eur Heart J.

[B23] Kersten JR, Pagel PS, Chilian WM, Warltier DC (1999). Multifactorial basis for coronary collateralization: a complex adaptive response to ischemia. Cardiovasc Res.

[B24] D Masuda RNTHKKLGCRHMIETMFSS (2001). Enhanced external counterpulsation improved myocardial perfusion and coronary flow reserve in patients with chronic stable angina. Evaluation by13N-ammonia positron emission tomography. Eur Heart J.

[B25] Lawson WE, Kennard ED, Hui JC, Holubkov R, Kelsey SF (2003). Analysis of baseline factors associated with reduction in chest pain in patients with angina pectoris treated by enhanced external counterpulsation. Am J Cardiol.

[B26] Lawson WE, Hui JC, Kennard ED, Barsness G, Kelsey SF (2005). Predictors of benefit in angina patients one year after completing enhanced external counterpulsation: initial responders to treatment versus nonresponders. Cardiology.

[B27] Niebauer J, Cooke JP (1996). Cardiovascular effects of exercise: role of endothelial shear stress. J Am Coll Cardiol.

[B28] Bonetti PO, Barsness GW, Keelan PC, Schnell TI, Pumper GM, Kuvin JT, Schnall RP, Holmes DR, Higano ST, Lerman A (2003). Enhanced external counterpulsation improves endothelial function in patients with symptomatic coronary artery disease. J Am Coll Cardiol.

[B29] Arora RR, Chou TM, Jain D, Fleishman B, Crawford L, McKiernan T, Nesto R, Ferrans CE, Keller S (2002). Effects of enhanced external counterpulsation on Health-Related Quality of Life continue 12 months after treatment: a substudy of the Multicenter Study of Enhanced External Counterpulsation. J Investig Med.

